# Identification of the amino acid residues involved in the species-dependent differences in the pyridoxine transport function of SLC19A3

**DOI:** 10.1016/j.jbc.2022.102161

**Published:** 2022-06-17

**Authors:** Kohei Miyake, Tomoya Yasujima, Syunsuke Takahashi, Takahiro Yamashiro, Hiroaki Yuasa

**Affiliations:** Department of Biopharmaceutics, Graduate School of Pharmaceutical Sciences, Nagoya City University, Mizuho-ku, Nagoya, Japan

**Keywords:** SLC19A3, SLC19A2, transporter, pyridoxine, species difference, vitamin, thiamine, cDNA, complementary DNA, EGFP, enhanced GFP, HEK293, human embryonic kidney 293 cell line, hSLC19A3, human solute carrier SLC19A3, hTMD, TMD in hSLC19A3, mSlc19a3, mouse Slc19a3, mTMD, TMD in mSlc19a3, TMD, transmembrane domain

## Abstract

Recent studies have shown that human solute carrier SLC19A3 (hSLC19A3) can transport pyridoxine (vitamin B6) in addition to thiamine (vitamin B1), its originally identified substrate, whereas rat and mouse orthologs of hSLC19A3 can transport thiamine but not pyridoxine. This finding implies that some amino acid residues required for pyridoxine transport, but not for thiamine transport, are specific to hSLC19A3. Here, we sought to identify these residues to help clarify the unique operational mechanism of SLC19A3 through analyses comparing hSLC19A3 and mouse Slc19a3 (mSlc19a3). For our analyses, hSLC19A3 mutants were prepared by replacing selected amino acid residues with their counterparts in mSlc19a3, and mSlc19a3 mutants were prepared by substituting selected residues with their hSLC19A3 counterparts. We assessed pyridoxine and thiamine transport by these mutants in transiently transfected human embryonic kidney 293 cells. Our analyses indicated that the hSLC19A3-specific amino acid residues of Gln^86^, Gly^87^, Ile^91^, Thr^93^, Trp^94^, Ser^168^, and Asn^173^ are critical for pyridoxine transport. These seven amino acid residues were found to be mostly conserved in the SLC19A3 orthologs that can transport pyridoxine but not in orthologs that are unable to transport pyridoxine. In addition, these residues were also found to be conserved in several SLC19A2 orthologs, including rat, mouse, and human orthologs, which were all found to effectively transport both pyridoxine and thiamine, exhibiting no species-dependent differences. Together, these findings provide a molecular basis for the unique functional characteristics of SLC19A3 and also of SLC19A2.

Recent work has shown that the human orthologs of SLC19A2 (hSLC19A2) and SLC19A3 (hSLC19A3) can transport pyridoxine (vitamin B6) in addition to their originally identified substrate, thiamine (vitamin B1) (1). Based on this finding, these transporters, known as thiamine transporter 1 and thiamine transporter 2, respectively, can now be recognized as pyridoxine/thiamine transporters. Because pyridoxine and thiamine are both basic compounds (Fig. 1), their cationic characteristics may contribute to their being recognized as substrates by these transporters. Notably, these transporters are the first pyridoxine transporters identified in humans. The physiological functions of SLC19A2 and SLC19A3 are indicated to be involved in the intestinal absorption of thiamine by operating for basolateral efflux and brush border uptake, respectively, in epithelial cells (2, 3, 4, 5). Hence, they could also play a role in pyridoxine absorption.

**Figure 1:**
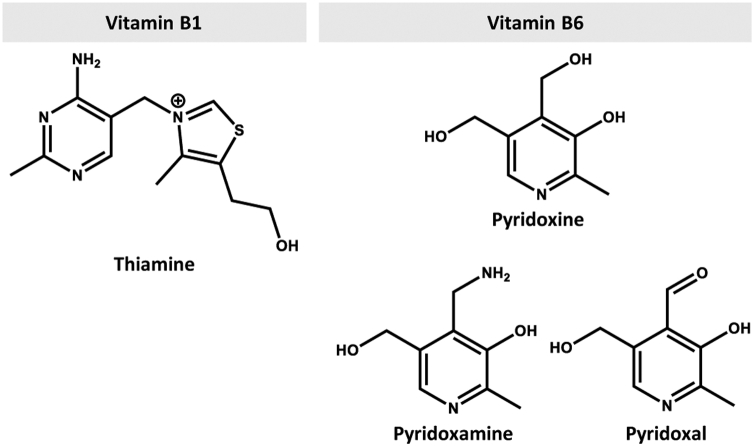
**Chemical structures of thiamine (vitamin B1) and pyridoxine (vitamin B6).** The structures of pyridoxamine and pyridoxal, the other two compounds that belong to vitamin B6, are also shown for reference.

However, other work has shown that SLC19A3s, which operate in brush border uptake, of rat and mouse lack the pyridoxine transport function (6). Consistent with this finding, pyridoxine uptake was low without any carrier-mediated characteristics in the everted tissue sacs of the rat small intestine (6), whereas involvement of hSLC19A3 in pyridoxine uptake was demonstrated in the Caco-2 cell line, a human intestinal epithelial cell model (1, 7). The SLC19A3s in those rodents can, on the other hand, transport thiamine, like hSLC19A3 (6). The inability to transport pyridoxine in those rodent orthologs suggests that they lack some amino acid residues present in hSLC19A3 required for pyridoxine but not thiamine, transport.

In the present study, we sought to identify hSLC19A3-specific amino acid residues required for the pyridoxine transport through analyses based on comparison of hSLC19A3 and mouse Slc19a3 (mSlc19a3). Physiological or pathological events that affect specific amino acid residues could potentially alter pyridoxine transport without affecting thiamine transport. Identifying such amino acid residues could help clarify the unique molecular and functional characteristics of hSLC19A3 and probe into related physiological and pathological issues.

Genetic defects in hSLC19A3 have been implicated in biotin–thiamine-responsive basal ganglia disease and Leigh syndrome (8, 9, 10), both serious neurological disorders. However, the pathological mechanisms involving hSLC19A3 have remained unclear, and thiamine supplementation has not always been an effective treatment for these disorders. Therefore, the newly found pyridoxine transport function of hSLC19A3 could be of interest as it may play a pathological role in these disorders. Similarly, the pyridoxine transport function of hSLC19A2 could be of interest because of its potential pathological role in thiamine-responsive megaloblastic anemia syndrome, a neurological disorder linked to genetic defects in hSLC19A2 (11, 12, 13). Notably, pyridoxine and thiamine deficiency have both been suggested to play a role in the development of neurological disorders. Pyridoxine plays an important role, typically in the form of its derived pyridoxal phosphate, as a cofactor in amino acid metabolism, and pyridoxine deficiency can cause various abnormalities, including microcytic anemia, dermatitis, and neurological disorders (14, 15). Similarly, thiamine serves, in the form of thiamine pyrophosphate, as a coenzyme in carbohydrate metabolism, and thiamine deficiency can cause various abnormalities including neurological and cardiovascular disorders (5).

## Results

### Identification of the transmembrane domains specifically involved in pyridoxine transport by hSLC19A3

**Figure 2:**
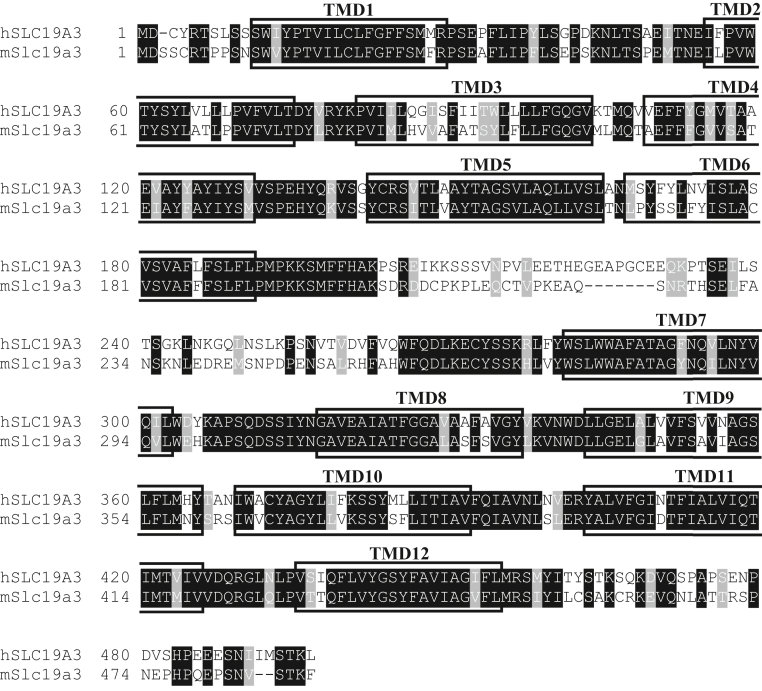
**Alignment of the amino acid sequence of hSLC19A3 with that of mSlc19a3.** The amino acid sequence of hSLC19A3 was aligned with that of mSlc19a3 using ClustalW program and visualized using BOXSHADE program. Conservatively different amino acid residues are indicated by *shading*. TMDs are indicated by *rectangles*. hSLC19A3, human solute carrier SLC19A3; mSlc19a3, mouse Slc19a3; TMD, transmembrane domain.

We first conducted an alignment analysis of amino acid sequences, comparing hSLC19A3, which can transport pyridoxine, with mSlc19a3, which cannot, to search for amino acid residues specifically required for pyridoxine transport by hSLC19A3. We identified amino acid residues specific to hSLC19A3 that are differed from their mSlc19a3 counterparts. As shown in Figure 2, our alignment analysis indicated that hSLC19A3, which is 69% identical to mSlc19a3, has as many as 160 hSLC19A3-specific amino acid residues. To narrow down candidate amino acid residues involved in pyridoxine transport, we focused on transmembrane domains (TMDs), which generally play important roles in substrate recognition and translocation. We examined the effect on pyridoxine transport by introducing each TMD in mSlc19a3 (mTMD) into hSLC19A3 (hTMD). Replacement involved replacement of all hSLC19A3-specific amino acid residues in the designated TMD with their mSlc19a3 counterparts.

Throughout the remaining analyses, assessments of pyridoxine uptake were conducted for an initial 2-min period at its trace concentration of 5 nM and pH 5.5 after transient transfection of a designated transporter or mutant into human embryonic kidney 293 (HEK293) cells. The uptake conditions were previously demonstrated to allow efficient pyridoxine transport in several SLC19A2 and SLC19A3 orthologs (1, 6). Assessments of thiamine uptake were also performed for comparison with the initial 2-min period at 5 nM and pH 7.4. This pH condition was selected because SLC19A2/3 orthologs favor near neutral conditions rather than acidic conditions for thiamine transport (1, 6). The specific uptake of each substrate was evaluated by subtracting the uptake in mock cells from that in transfected cells.

**Figure 3:**
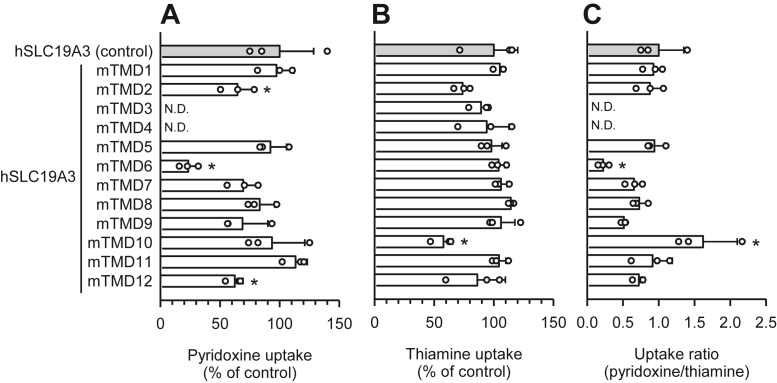
**Uptake of pyridoxine and thiamine by hSLC19A3 mutants in transiently transfected HEK293 cells: the effect of the introduction of mTMDs.** All the transporters and mutants were tagged with EGFP at the N terminus. *A*, the specific uptake of [^3^H]pyridoxine (5 nM) was evaluated for 2 min at pH 5.5 and 37 °C. *B*, the specific uptake of [^3^H]thiamine (5 nM) was evaluated for 2 min at pH 7.4 and 37 °C. *C*, the uptake ratio was calculated by dividing pyridoxine uptake by thiamine uptake, using uptake values in percent of control. The uptake of pyridoxine and thiamine by hSLC19A3 as a control was 47.5 and 40.8 fmol/min/mg protein, respectively. Data are presented as means ± SD (n = 3). ∗*p* < 0.05 compared with control. EGFP, enhanced GFP; HEK293, human embryonic kidney 293 cell line; hSLC19A3, human solute carrier SLC19A3; mTMD, TMD in mSlc19a3; ND, not detected; TMD, transmembrane domain.

Among the 12 mTMDs, mTMDs 2, 3, 4, 6, and 12 caused significant reductions in the specific uptake of pyridoxine when introduced into hSLC19A3, compared with pyridoxine uptake by the wildtype hSLC19A3 (Fig. 3*A*). The specific uptake of thiamine was, on the other hand, maintained at levels greater than 50% by hSLC19A3 in all the mutants, with a significant reduction caused only by introduction of mTMD10 (Fig. 3*B*). Based on these data, we calculated the ratio of pyridoxine uptake to thiamine uptake (pyridoxine/thiamine uptake ratio) to correct for the modest variation in thiamine uptake and used the ratio as a measure for the evaluation of the pyridoxine uptake activity. The uptake ratio was, as shown in Figure 3*C*, significantly reduced only in the hSLC19A3 mutants that had mTMD 3, 4, or 6 replacements. These results indicate that hTMDs 3, 4, and 6 could be specifically involved in the pyridoxine transport function of hSLC19A3.

Genetically modifying a transporter may cause an alteration in its protein expression level at the plasma membrane, which could in turn affect apparent uptake activity. However, the uptake ratio is independent of protein expression level, as the ratio represents relative activity for pyridoxine uptake normalized to thiamine uptake for the same transporter or modulated protein and, hence, for the same protein expression level. Therefore, analysis of pyridoxine uptake activity is possible without considering protein expression level, using the relative activity. Notably, the TMD replacements had no major impacts on thiamine uptake. This result may suggest that, as the thiamine uptake activity of mSlc19a3 was comparable to that of hSLC19A3, the intrinsic activity for thiamine uptake was mostly unchanged, together with protein expression. It is possible that the modest changes observed in thiamine uptake represent variation at the protein expression level mostly caused by technical variation in transfection efficiencies. Under the assumption, the changes in relative activity could reflect changes in intrinsic activity for pyridoxine uptake quite well. We also used relative activity, while confirming that thiamine uptake activity was mostly unaffected, for evaluating pyridoxine uptake in subsequent analyses.

**Figure 4:**
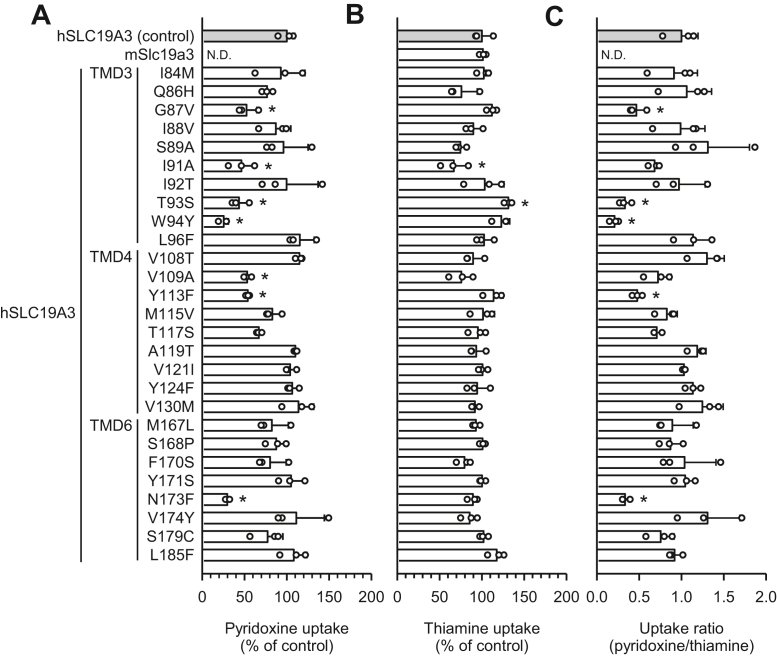
**Uptake of pyridoxine and thiamine by hSLC19A3 mutants in transiently transfected HEK293 cells: the effect of replacing hSLC19A3-specific amino acid residues in hTMDs 3, 4, and 6 with mSlc19a3 counterparts.** All the transporters and mutants were tagged with EGFP at the N terminus. *A*, the specific uptake of [^3^H]pyridoxine (5 nM) was evaluated for 2 min at pH 5.5 and 37 °C. *B*, the specific uptake of [^3^H]thiamine (5 nM) was evaluated for 2 min at pH 7.4 and 37 °C. *C*, the uptake ratio was calculated by dividing pyridoxine uptake by thiamine uptake, using uptake values in percent of control. The uptake of pyridoxine and thiamine by hSLC19A3 as a control was 36.6 and 50.0 fmol/min/mg protein, respectively. Data are presented as means ± SD (n = 3). ∗*p* < 0.05 compared with control. EGFP, enhanced GFP; HEK293, human embryonic kidney 293 cell line; hSLC19A3, human solute carrier SLC19A3; hTMD, TMD in hSLC19A3; mSlc19a3, mouse Slc19a3; ND, not detected; TMD, transmembrane domain.

**Figure 5:**
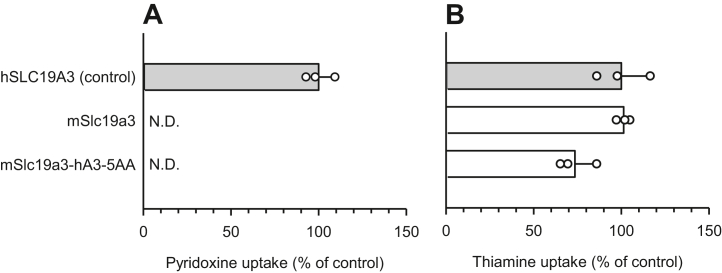
**Uptake of pyridoxine and thiamine by mSlc19a3-hA3-5AA in transiently transfected HEK293 cells.** The mSlc19a3 mutant of mSlc19a3-hA3-5AA was prepared by introducing the five hSLC19A3-specific amino acid residues suggested to be involved in the pyridoxine transport function (Fig. 4) in place of their mSlc19a3 counterparts. All the transporters and mutants were tagged with EGFP at the N terminus. *A*, the specific uptake of [^3^H]pyridoxine (5 nM) was evaluated for 2 min at pH 5.5 and 37 °C. *B*, the specific uptake of [^3^H]thiamine (5 nM) was evaluated for 2 min at pH 7.4 and 37 °C. The uptake of pyridoxine and thiamine by hSLC19A3 as a control was 46.6 and 58.8 fmol/min/mg protein, respectively. Data are presented as means ± SD (n = 3). EGFP, enhanced GFP; HEK293, human embryonic kidney 293 cell line; hSLC19A3, human solute carrier SLC19A3; mSlc19a3, mouse Slc19a3; ND, not detected.

Next, we prepared hSLC19A3 mutants in which each hSLC19A3-specific amino acid residue in hTMDs 3, 4, and 6 was replaced with its mSlc19a3 counterpart in order to identify the residues involved in pyridoxine transport (Fig. 4). We found that the mutations G87V, T93S, W94Y, Y113F, and N173F lead to significant reductions in the pyridoxine/thiamine uptake ratio (Fig. 4*C*). These results suggest the involvement of Gly^87^, Thr^93^, Trp^94^, Tyr^113^, and Asn^173^ in pyridoxine transport. In almost all mutants, the thiamine uptake activity was maintained without any significant alteration, with the exceptions of a slight decrease in thiamine uptake by I91A and a slight increase in thiamine uptake by T93S (Fig. 4*B*). However, introduction of all five hSLC19A3-specific residues together, replacing their mSlc19a3 counterparts, could not confer the pyridoxine transport function to mSlc19a3 (Fig. 5*A*), indicated by the undetectable pyridoxine uptake activity of the new mSlc19a3 mutant (mSlc19a3-hA3-5AA) while thiamine uptake activity was unchanged (Fig. 5*B*). Therefore, additional residues may be required for mSlc19a3 to acquire the pyridoxine transport function.

**Figure 6:**
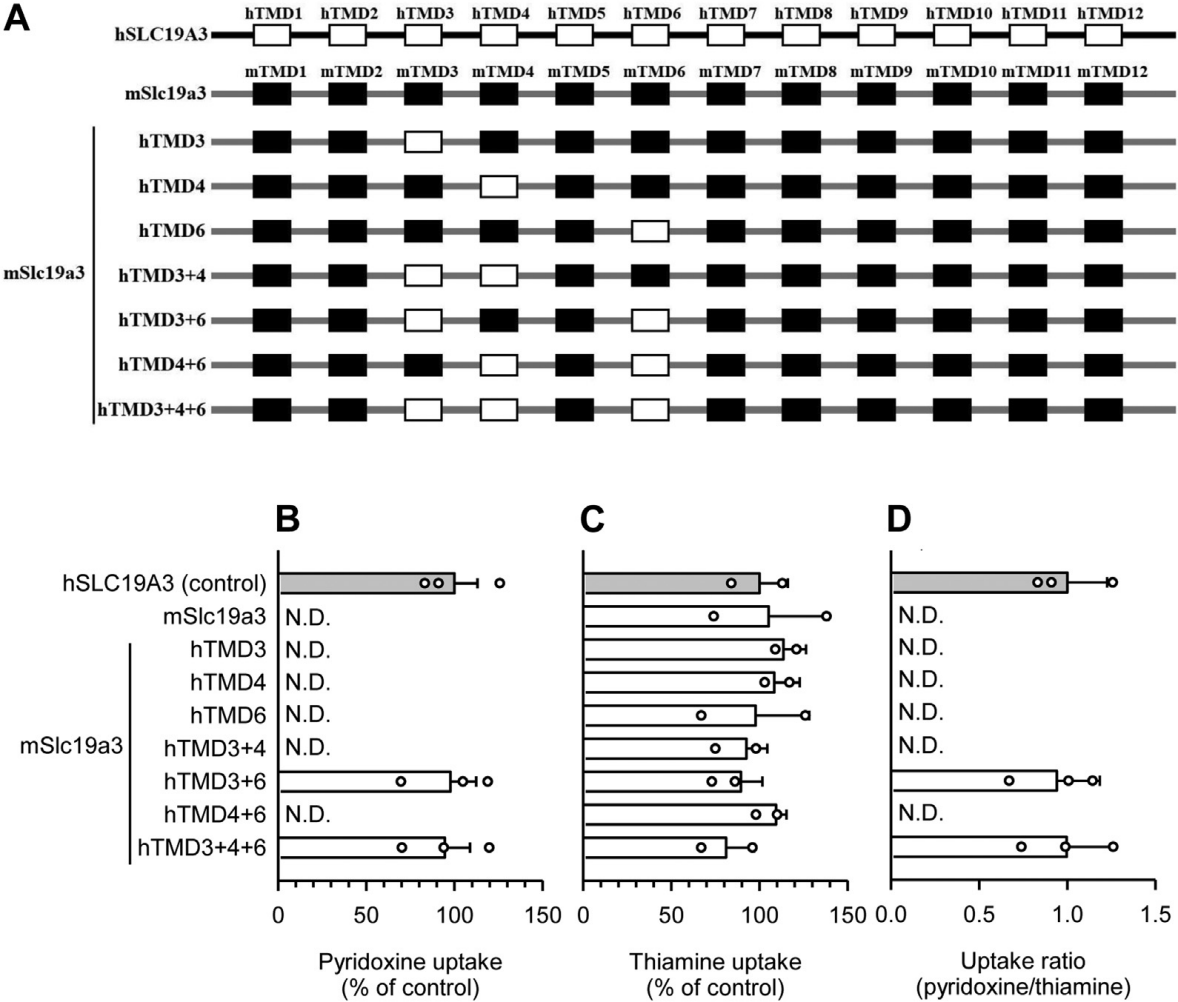
**Uptake of pyridoxine and thiamine by mSlc19a3 mutants in transiently transfected HEK293 cells: the effect of the introduction of hTMDs.***A*, schematic representation of mSlc19a3 mutants that have single or multiple hTMDs in place of their mTMD counterparts. All the transporters and mutants were tagged with EGFP at the N terminus. *B*, the specific uptake of [^3^H]pyridoxine (5 nM) was evaluated for 2 min at pH 5.5 and 37 °C. *C*, the specific uptake of [^3^H]thiamine (5 nM) was evaluated for 2 min at pH 7.4 and 37 °C. *D*, the uptake ratio was calculated by dividing pyridoxine uptake by thiamine uptake, using uptake values in percent of control. The uptake of pyridoxine and thiamine by hSLC19A3 as a control was 58.5 and 50.2 fmol/min/mg protein, respectively. Data are presented as means ± SD (n = 3). EGFP, enhanced GFP; HEK293, human embryonic kidney 293 cell line; hTMD, TMD in hSLC19A3; mSlc19a3, mouse Slc19a3; mTMD, TMD in mSlc19a3; ND, not detected; TMD, transmembrane domain.

To explore alternative strategies to determine requirements for pyridoxine transport, we examined whether the introduction of hTMDs 3, 4, and 6 into mSlc19a3, replacing their counterpart mTMDs, could confer the pyridoxine transport function (Fig. 6*A*). As shown in Figure 6, *B*–*D*, introduction of all three hTMDs together conferred the pyridoxine transport function to mSlc19a3 at an uptake activity comparable to that of hSLC19A3, as indicated by uptake of both pyridoxine and thiamine by the mSlc19a3 mutant (mSlc19a3-hTMD3+4+6) at levels comparable to respective uptake levels by hSLC19A3 and, accordingly, a pyridoxine/thiamine uptake ratio of almost one. Therefore, this mSlc19a3 mutant, which was similar to hSLC19A3 in that it had all three hTMDs implicated in pyridoxine transport, acquired the pyridoxine transport function. Pyridoxine transport was absent in the mSlc19a3 mutants in which hTMDs 3 and 4 were introduced together (mSlc19a3-hTMD3+4) and in which hTMDs 4 and 6 were introduced together (mSlc19a3-hTMD4+6). In the former, mTMD6 was present in place of hTMD6 in mSlc19a3-hTMD3+4+6 and, in the latter, mTMD3 was present in place of hTMD3. These results were in agreement with the nearly complete lack of pyridoxine transport after introduction of each of mTMDs 3 and 6 into hSLC19A3 (Fig. 3*C*). However, the mSlc19a3 mutant in which hTMDs 3 and 6 were introduced together (mSlc19a3-hTMD3+6), mTMD4 being present in place of hTMD4 in mSlc19a3-hTMD3+4+6, exhibited a pyridoxine uptake activity comparable to those of hSLC19A3 and mSlc19a3-hTMD3+4+6, suggesting that hTMD4 does not play a role in conferring the pyridoxine transport function to mSlc19a3, although the introduction of mTMD4 into hSLC19A3, replacing hTMD4, prevented pyridoxine transport (Fig. 3*C*). In addition, the introduction of hTMD 3, 4, or 6 alone did not confer the pyridoxine transport function to mSlc19a3. Taken together, these results suggest that hTMDs 3 and 6 are required for pyridoxine transport. In addition, while hTMD4 may not be essential for pyridoxine transport, mTMD4 can disturb pyridoxine transport when introduced into hSLC19A3 in place of hTMD4. Notably, thiamine uptake activity was not altered by any of the modulations (Fig. 6*C*).

### Identification of the amino acid residues required for pyridoxine transport in hSLC19A3

**Figure 7:**
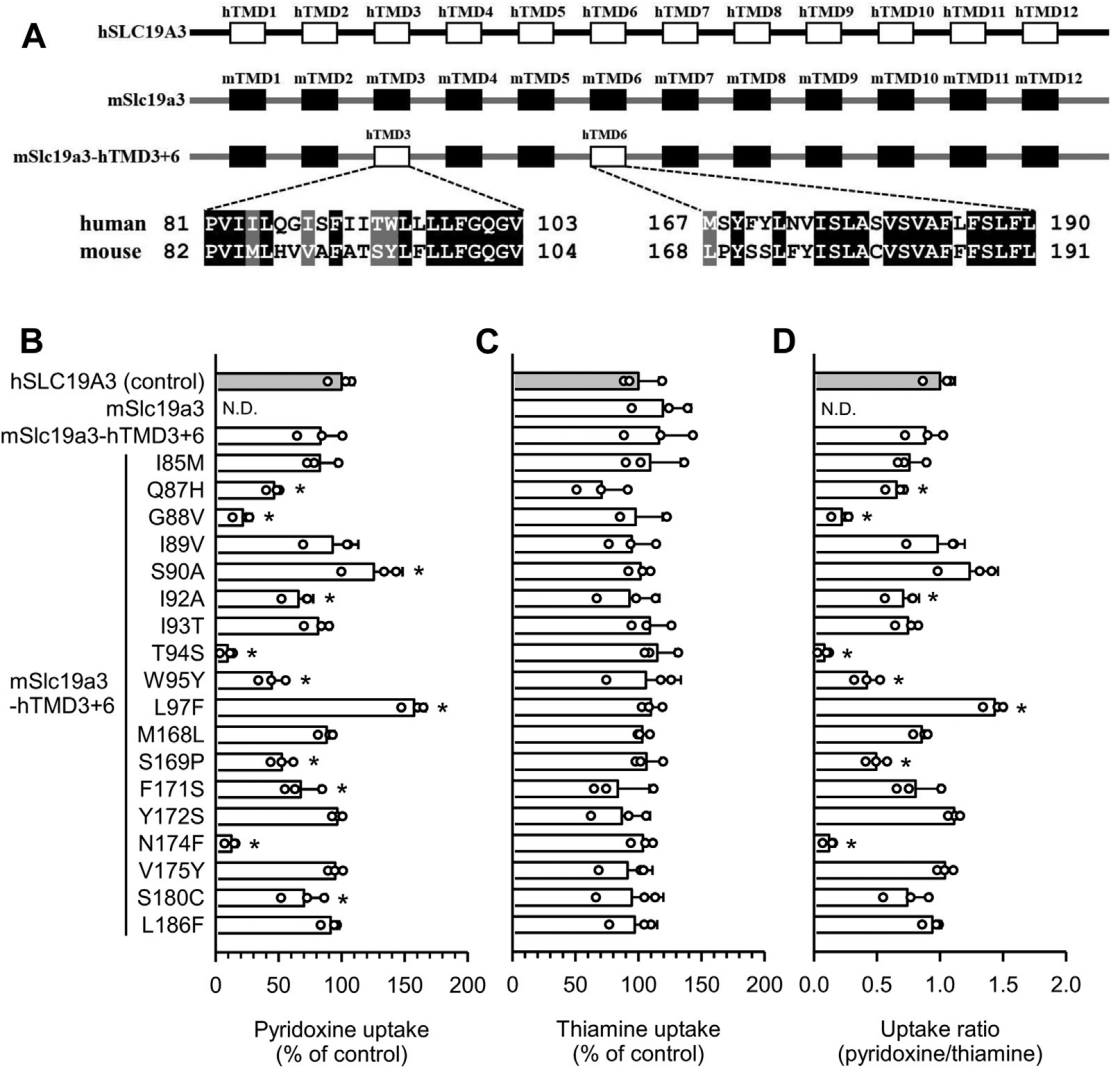
**Uptake of pyridoxine and thiamine by various mutants derived from mSlc19a3-hTMD3+6 in transiently transfected HEK293 cells.***A*, schematic representation of various mutants derived from mSlc19a3-hTMD3+6, the mSlc19a3 mutant that has hTMDs 3 and 6 together in place of their respective mTMD counterparts. All the transporters and mutants were tagged with EGFP at the N terminus. *B*, the specific uptake of [^3^H]pyridoxine (5 nM) was evaluated for 2 min at pH 5.5 and 37 °C. *C*, the specific uptake of [^3^H]thiamine (5 nM) was evaluated for 2 min at pH 7.4 and 37 °C. *D*, the uptake ratio was calculated by dividing pyridoxine uptake by thiamine uptake, using uptake values in percent of control. The uptake of pyridoxine and thiamine by hSLC19A3 as a control was 46.3 and 62.6 fmol/min/mg protein, respectively. Data are presented as means ± SD (n = 3). ∗*p* < 0.05 compared with control. EGFP, enhanced GFP; HEK293, human embryonic kidney 293 cell line; hSLC19A3, human solute carrier SLC19A3; hTMD, TMD in hSLC19A3; mSlc19a3, mouse Slc19a3; mTMD, TMD in mSlc19a3; ND, not detected; TMD, transmembrane domain.

We proceeded to identify the amino acid residues required for pyridoxine transport using the mSlc19a3-hTMD3+6 mutant and its derived mutants, in which each hSLC19A3-specific amino acid residue in hTMDs 3 and 6 was replaced with its mSlc19a3 counterpart (Fig. 7*A*). As shown in Figure 7, *B*–*D*, the mutations Q87H, G88V, I92A, T94S, W95Y, S169P, and N174F led to significant reductions in pyridoxine uptake activity, suggesting that Gln^86^, Gly^87^, Ile^91^, Thr^93^, Trp^94^, Ser^168^, and Asn^173^, which are the hSLC19A3 residues corresponding to the mutated ones, could be critical for the pyridoxine transport function in hSLC19A3. Compared with the five residues initially suggested in Figure 4, Gln^86^ and Ile^91^ in hTMD3 and Ser168 in hTMD6 were also suggested to be required, whereas Tyr^113^ in hTMD4 was not required. The thiamine uptake activity was maintained without any significant changes in all mutants (Fig. 7*C*).

**Figure 8:**
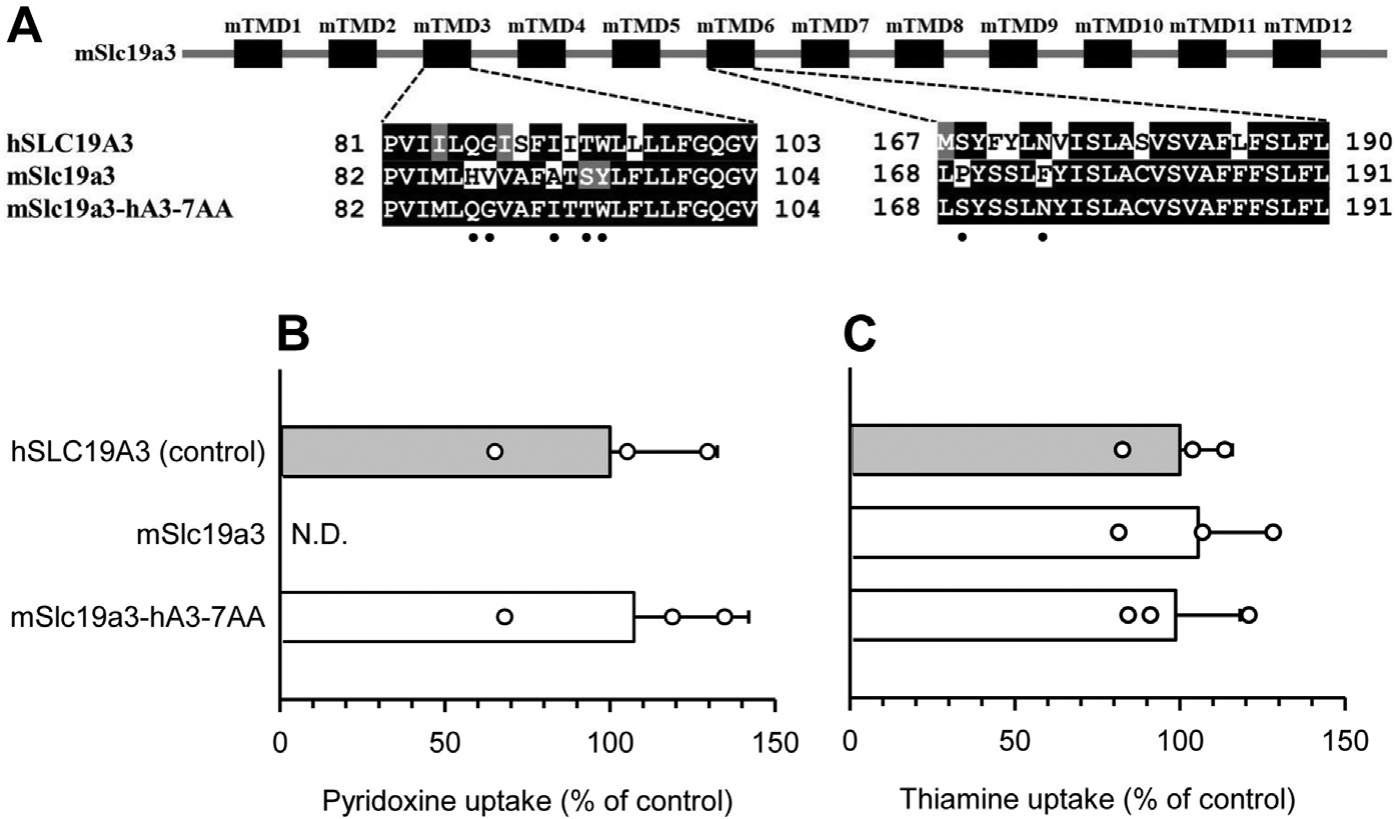
**Uptake of pyridoxine and thiamine by mSlc19a3-hA3-7AA in transiently transfected HEK293 cells.***A*, schematic representation of mSlc19a3-hA3-7AA, the mSlc19a3 mutant that has the seven hSLC19A3-derived amino acid residues required for the pyridoxine transport function (Fig. 7) in place of their mSlc19a3 counterparts. The residues are indicated by *dots*. All the transporters and mutants were tagged with EGFP at the N terminus. *B*, the specific uptake of [^3^H]pyridoxine (5 nM) was evaluated for 2 min at pH 5.5 and 37 °C. *C*, the specific uptake of [^3^H]thiamine (5 nM) was evaluated for 2 min at pH 7.4 and 37 °C. The uptake of pyridoxine and thiamine by hSLC19A3 as a control was 69.8 and 65.1 fmol/min/mg protein, respectively. Data are presented as means ± SD (n = 3). EGFP, enhanced GFP; HEK293, human embryonic kidney 293 cell line; hSLC19A3, human solute carrier SLC19A3; mSlc19a3, mouse Slc19a3; ND, not detected.

To confirm the role of the seven hSLC19A3-specific amino acid residues in pyridoxine transport, we prepared an mSlc19a3 mutant that had all seven residues in place of their mSlc19a3 counterparts (mSlc19a3-hA3-7AA) (Fig. 8*A*). As shown in Figure 8*B*, the specific uptake of pyridoxine by the mutant was comparable to uptake by hSLC19A3, indicating that the mutant fully gained the ability to transport pyridoxine because of the presence of the seven amino acid residues. We confirmed that thiamine uptake activity was unchanged in the mutant (Fig. 8*C*).

**Figure 9:**
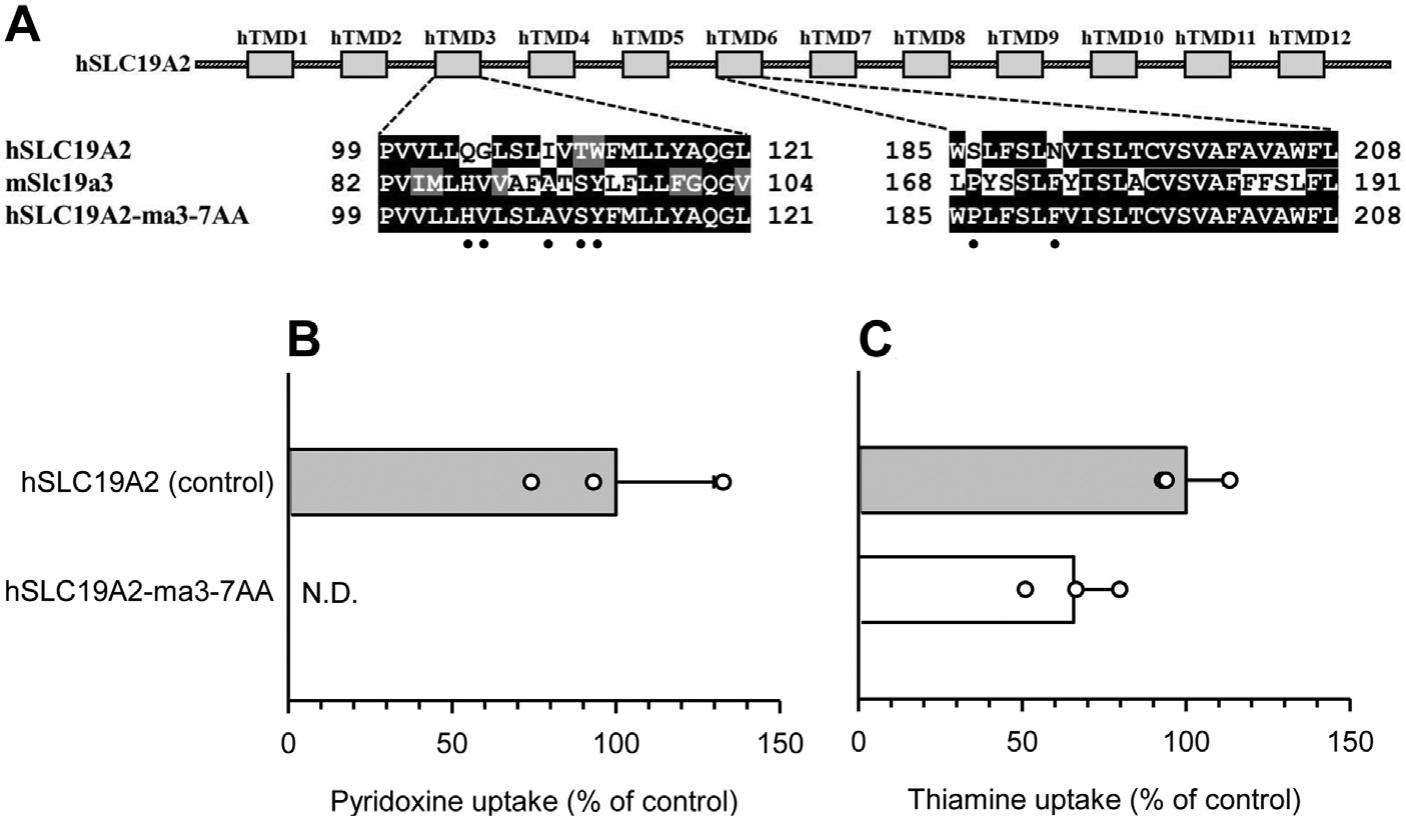
**Uptake of pyridoxine and thiamine by hSLC19A2-ma3-7AA in transiently transfected HEK293 cells.***A*, schematic representation of hSLC19A2-ma3-7AA, the hSLC19A2 mutant in which the seven amino acid residues corresponding to the hSLC19A3 residues required for the pyridoxine transport function (Fig. 7) are replaced with their mSlc19a3 counterparts. The residues are indicated by *dots*. hSLC19A2 and the mutant were tagged with EGFP at the N terminus. *B*, the specific uptake of [^3^H]pyridoxine (5 nM) was evaluated for 2 min at pH 5.5 and 37 °C. *C*, the specific uptake of [^3^H]thiamine (5 nM) was evaluated for 2 min at pH 7.4 and 37 °C. The uptake of pyridoxine and thiamine by hSLC19A2 as a control was 36.6 and 70.5 fmol/min/mg protein, respectively. Data are presented as means ± SD (n = 3). EGFP, enhanced GFP; HEK293, human embryonic kidney 293 cell line; hSLC19A2, human solute carrier SLC19A3; mSlc19a3, mouse Slc19a3; ND, not detected.

Notably, TMDs 3 and 6 of hSLC19A2 are structurally similar to those in hSLC19A3, in that they have the critical seven amino acid residues at the corresponding positions (Fig. 9*A*). Therefore, we prepared an hSLC19A2 mutant in which all seven residues were replaced with their mSlc19a3 counterparts (hSLC19A2-ma3-7AA) and examined pyridoxine transport by the mutant. As expected, the specific uptake of pyridoxine was reduced to an undetectable level in the mutant, indicating that pyridoxine transport was eliminated by introduction of those mSlc19a3-derived residues (Fig. 9*B*). The thiamine uptake activity was, on the other hand, unchanged in the mutant (Fig. 9*C*). This finding further supports the proposed role of the seven amino acid residues in the pyridoxine transport function.

### Comparative assessments of pyridoxine transport by the SLC19A3s and SLC19A2s of selected animal species

**Figure 10:**
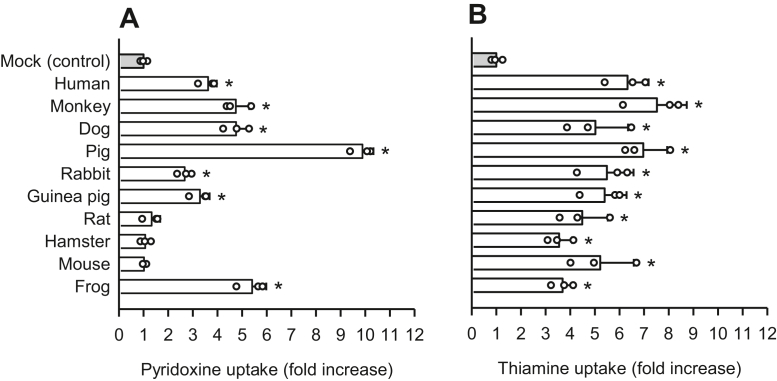
**Uptake of pyridoxine and thiamine in transiently transfected HEK293 cells expressing the SLC19A3s of selected animal species.***A*, the uptake of [^3^H]pyridoxine (5 nM) was evaluated for 2 min at pH 5.5 and 37 °C in cells expressing the SLC19A3s of selected animal species. *B*, the uptake of [^3^H]thiamine (5 nM) was similarly evaluated for 2 min at pH 7.4 and 37 °C. The uptake of pyridoxine and thiamine in mock cells as a control was 11.0 and 8.5 fmol/min/mg protein, respectively. No transporters had the EGFP tag. Data are presented as means ± SD (n = 3). ∗*p* < 0.05 compared with control. EGFP, enhanced GFP; HEK293, human embryonic kidney 293 cell line; SLC19A3, solute carrier SLC19A3.

**Figure 11:**
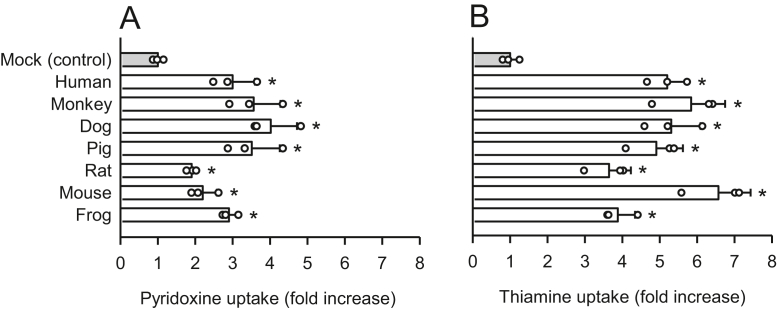
**Uptake of pyridoxine and thiamine in transiently transfected HEK293 cells expressing the SLC19A2s of selected animal species.***A*, the uptake of [^3^H]pyridoxine (5 nM) was evaluated for 2 min at pH 5.5 and 37 °C in cells expressing the SLC19A2s of selected animal species. *B*, the uptake of [^3^H]thiamine (5 nM) was similarly evaluated for 2 min at pH 7.4 and 37 °C. The uptake of pyridoxine and thiamine in mock cells for control was 15.0 and 14.4 fmol/min/mg protein, respectively. No transporters had the EGFP tag. Data are presented as means ± SD (n = 3). ∗*p* < 0.05 compared with control. EGFP, enhanced GFP; HEK293, human embryonic kidney 293 cell line; SLC19A2, solute carrier SLC19A2.

Following the identification of the seven critical amino acid residues required for pyridoxine transport in hSLC19A3 and also in hSLC19A2, we assessed the SLC19A3s and SLC19A2s of several animal species to determine conservation of those amino acid residues as it relates to the pyridoxine transport function. When introduced into HEK293 cells, the SLC19A3s of monkey, dog, pig, rabbit, guinea pig, and frog, as well as human, induced increases in pyridoxine uptake, compared with uptake in mock cells, whereas the SLC19A3s of rat, hamster, and mouse did not (Fig. 10*A*). Thiamine uptake, on the other hand, increased in all SLC19A3 orthologs (Fig. 10*B*). Among SLC19A2 orthologs, monkey, dog, pig, rat, mouse, and frog all induced increases in pyridoxine uptake, in addition to the human ortholog (Fig. 11*A*). Thiamine uptake was also increased by introduction of all SLC19A2 orthologs (Fig. 11*B*). Together, these results indicate that only the SLC19A3s of rat, hamster, and mouse specifically lack the pyridoxine transport function.

**Figure 12:**
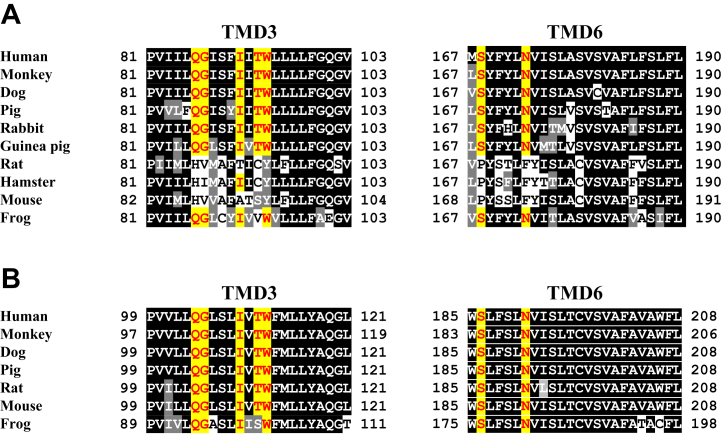
**Analysis of alignment of the amino acid sequences in TMD3 and TMD6 of the SLC19A3s and SLC19A2s of selected animal species.***A*, the amino acid sequences in the TMD3 and TMD6 of human SLC19A3 were aligned with those in the SLC19A3s of selected animal species using ClustalW program and visualized using BOXSHADE program. The amino acid residues required for the pyridoxine transport function are marked in *red*. Conservatively different amino acid residues are indicated by *shading*. *B*, the amino acid sequence in the TMD3 and TMD6 of human SLC19A2 was similarly aligned with those of the SLC19A2s of selected animal species. SLC19A, solute carrier SLC19A; TMD, transmembrane domain.

Accordingly, all seven critical amino acid residues were conserved in nearly all SLC19A2/3 orthologs with the pyridoxine transport function, with the exception of frog orthologs (Fig. 12). In the frog SLC19A3 and SLC19A2, a single amino acid residue of Thr^93^, among the seven residues, was not conserved and, instead, they had Val and Ser, respectively, in place of this residue. This implies that Val and Ser can play a role in the pyridoxine transport function in the absence of Thr. In the rat ortholog of SLC19A3, none of the seven residues were conserved, as in the mouse ortholog. In the hamster ortholog, six residues were not conserved; the exception was Ile^91^. Notably, there was an N-to-F substitution at position 173 in both the rat and hamster orthologs, which can almost completely deprive of the pyridoxine transport function, as demonstrated in Figure 7 (N174F in mSlc19a3-hTMD3+6).

## Discussion

We identified Gln^86^, Gly^87^, Ile^91^, Thr^93^, Trp^94^, Ser^168^, and Asn^173^ as the critical seven amino acid residues required for pyridoxine, but not thiamine, transport in hSLC19A3. The role of these residues in pyridoxine transport was further supported by the finding that these residues are mostly conserved in the SLC19A3 orthologs with the ability to transport pyridoxine but not in the orthologs that lack this function. Furthermore, these residues were found to be mostly conserved in all SLC19A2 orthologs examined in this study and, accordingly, they were all found to be able to transport pyridoxine. Therefore, it seems that SLC19A2/3 orthologs are generally capable of transporting pyridoxine, with the exception of a limited group of animal species SLC19A3s, including those of rat, mouse, and hamster. In addition, the pyridoxine transport function is absent specifically in SLC19A3, whereas it is retained in SLC19A2, in rat, mouse, and hamster. Although these SLC19As have quite similar transport functions and exhibit similarly ubiquitous expression profiles, they exhibit different subcellular localizations in polarized cells, with SLC19A3 being localized apically and SLC19A2 localized basolaterally (1, 2, 3, 4, 5, 16). Among such polarized cells are intestinal epithelial cells, in which the apically localized SLC19A3 could potentially play an important role in the absorption of pyridoxine by operating in its apical (brush border) uptake. It is intriguing that the deprivation of the pyridoxine transport function of SLC19A3 may have occurred in some particular animals in an adaptative process to lower the pyridoxine intake out of some, currently unknown biological or physiological necessity, while retaining its function of thiamine uptake. The pyridoxine transport function of SLC19A2 may be retained, as this transporter is not involved in apical uptake. Those animals in which SLC19A3-mediated pyridoxine transport is absent would indeed have poor ability for intestinal pyridoxine absorption, as demonstrated in rats (6), and they may be less dependent on pyridoxine from external sources. However, these species are still likely to have some pyridoxine in plasma owing to a low level of intestinal uptake by simple diffusion, as shown in rats (6, 15). Therefore, SLC19A2 could still play a role in pyridoxine disposition in those animals. There is also a possibility that unidentified pyridoxine transporter(s) might compensate for the absence of SLC19A3-mediated pyridoxine transport in pyridoxine disposition after absorption.

Notably, there are two other vitamin B6 compounds, pyridoxamine and pyridoxal (Fig. 1). Based on their inhibitory effects on hSLC19A3-mediated pyridoxine transport, it has been suggested that pyridoxamine has a higher affinity for hSLC19A3 over pyridoxine, but pyridoxal is poorly recognized by hSLC19A3 (1). Therefore, pyridoxamine could be an additional substrate transported by the same mechanism that transports pyridoxine and, hence, would not be absorbed by the SLC19A3-mediated mechanism in animals lacking pyridoxine transport by their SLC19A3 ortholog. Pyridoxal is, on the other hand, unlikely to be transported efficiently by SLC19A3. Therefore, it is possible that an unidentified transporter performs pyridoxal uptake in the intestine, and those animals might rely mainly on pyridoxal as a vitamin B6 compound supplied externally. Apart from that, animals in which the SLC19A3 ortholog lacks the pyridoxine transport function are not suitable as model animals for studies on the disposition of pyridoxine and related issues. Understanding the critical seven amino acid residues required for pyridoxine transport by SLC19A3 and also by SLC19A2 should help identify such animals.

**Figure 13:**
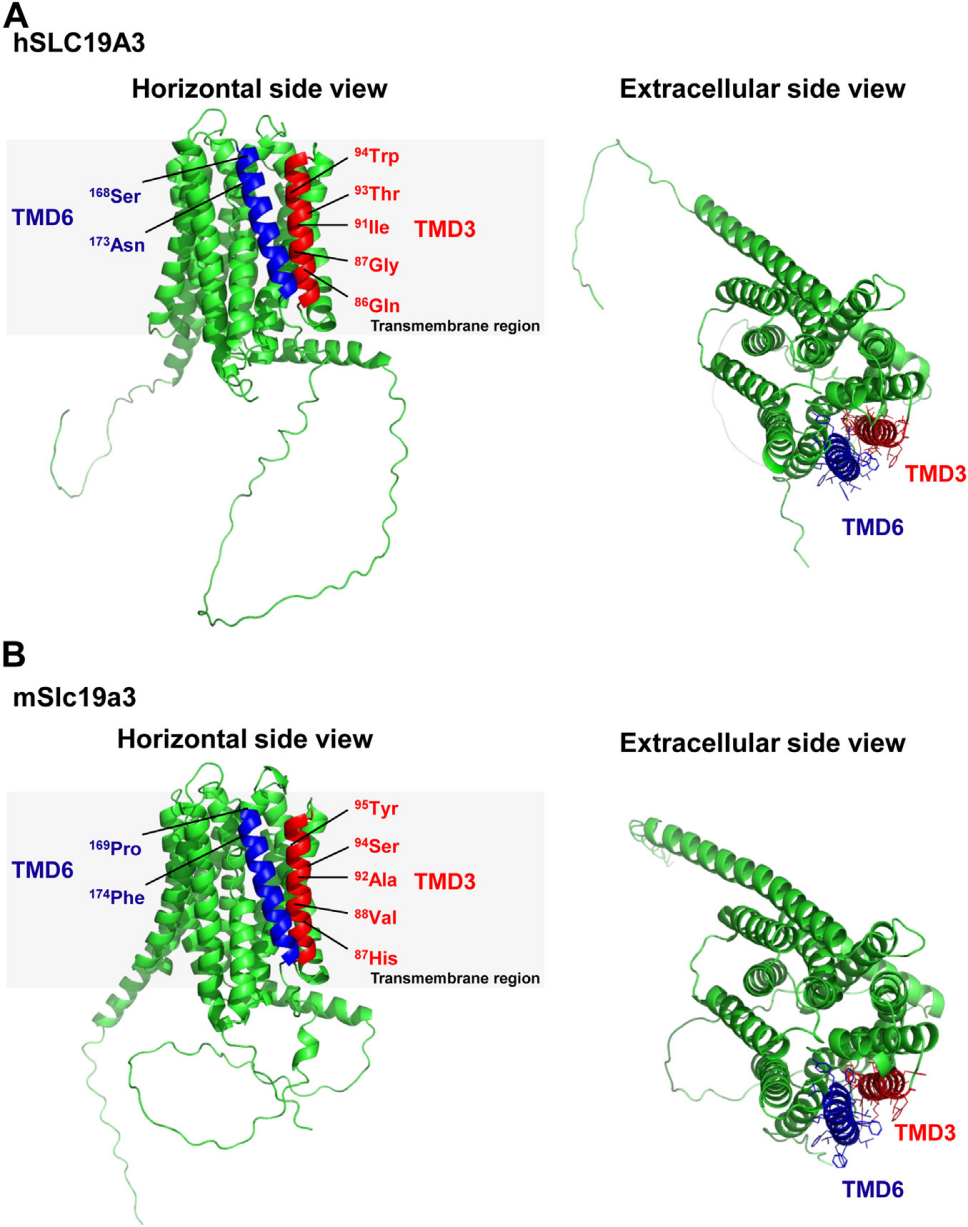
**Predicted structure of hSLC19A3.** The structures of hSLC19A3 (*A*) and mSlc19a3 (*B*) from AlphaFold Protein Structure Database (17) were visualized from the horizontal and extracellular sides with PyMol program to highlight TMD3 (*red*) and TMD6 (*blue*). The positions of the critical residues needed for the pyridoxine transport function (^86^Gln, ^87^Gly, ^91^Ile, ^93^Thr, and ^94^Trp in TMD3 and ^168^Ser and ^173^Asn in TMD6) in hSLC19A3 and their counterparts in mSlc19a3 are indicated. hSLC19A3, human solute carrier SLC19A3; mSlc19a3, mouse Slc19a3; TMD, transmembrane domain.

Although the specific roles of the critical seven amino acid residues required for the pyridoxine transport function of SLC19A2/3 remain unknown, all residues are, according to the structure of hSLC19A3 available from the AlphaFold Protein Structure Database (17) generated by AlphaFold version 2.0 AI system of DeepMind (18), located by a space adjunct to a large cavity that could be the putative substrate-binding site (Fig. 13*A*). Since mSlc19a3 appears similar to hSLC19A3 in structure (Fig. 13*B*), the differences in the amino acid residues may only impact interaction with substrates and not configuration of the space. The seven residues may also be involved in the uniquely different characteristics observed for pyridoxine transport compared with thiamine transport. As reported previously (1, 6), pyridoxine transport is highly dependent on pH, favoring acidic conditions, and likely to be coupled with H^+^, whereas thiamine transport is optimal under near neutral conditions, depending only moderately on pH. In addition, whereas thiamine inhibits pyridoxine transport competitively, pyridoxine inhibits thiamine transport noncompetitively (1). The critical residues may include those responsible for the preference for acidic pH in pyridoxine transport. Some or all the seven critical residues may also be involved in the recognition of pyridoxine but not of thiamine, and the presence of these extra residues for pyridoxine recognition may manifest in the noncompetitive inhibition of thiamine transport by pyridoxine. The amino acid residues required for the recognition of thiamine may be shared by those required for the recognition of pyridoxine, manifesting in the competitive inhibition of pyridoxine transport by thiamine. We also note the possibility that specific changes to the critical residues may lead to malfunctions of SLC19A2/3 only in their ability to transport pyridoxine, without affecting their ability to transport thiamine. Further studies are warranted in order to clarify the precise roles of these residues. In addition, future work should explore the possibility that the residues may constitute or include a consensus sequence that could have a similar function in analogous transporters.

hSLC19A2 and hSLC19A3 have been of interest because of their involvement in some genetic neurological disorders. Genetic dysfunction of hSLC19A2 has been linked to thiamine-responsive megaloblastic anemia (11, 12, 13), whereas genetic dysfunction of hSLC19A3 has been linked to biotin–thiamine-responsive basal ganglia disease and Leigh syndrome (8, 9, 10). Previous studies have explored links between the thiamine transport function of those hSLC19As and disease states, and thiamine supplementation has been a treatment for potential thiamine deficiency (19). Now that those hSLC19As have been found to operate dual-functionally in both pyridoxine transport and thiamine transport, potential alterations in the pyridoxine transport function should also be considered. Since the present study identified amino acid residues involved only in the pyridoxine transport function in those hSLC19As, genetic mutations could potentially have different impacts on pyridoxine transport and thiamine transport. Thus, genetic disorders linked to those hSLC19As may involve defects in the pyridoxine transport function in addition to, or rather than, defects in thiamine transport and, resultingly, pyridoxine deficiency. If that is indeed the case, pyridoxine supplementation could be considered as an additional or alternative treatment for these diseases.

In summary, we have successfully identified seven critical amino acid residues required for pyridoxine transport by SLC19A3. These residues were found to be well conserved in the SLC19A3 orthologs that have the pyridoxine transport function but not in those that do not. These residues were also found to be present and generally conserved in SLC19A2 orthologs, which do not exhibit animal species differences in that function. We further note that these residues could be involved in the uniquely different characteristics of SLC19A2/3 operation of pyridoxine transport and thiamine transport. In studies on the disposition of pyridoxine and related issues, model animals in which the SLC19A3 ortholog lacks the pyridoxine transport function or those in which the SLC19A2 lacks the function, if any, would not be suitable for use. The knowledge of these critical residues should be of help in identifying such animals.

## Experimental procedures

### Materials

[^3^H]Pyridoxine (20 Ci/mmol) and [^3^H]thiamine (20 Ci/mmol) were obtained from American Radiolabeled Chemicals. Dulbecco's modified Eagle's medium was obtained from Wako Pure Chemical Industries, and fetal bovine serum was obtained from Sigma–Aldrich. All other reagents were of analytical grade and commercially obtained.

### Cells and culture

HEK293, Madin–Darby canine kidney II, and Lilly Laboratories cell-porcine kidney 1 cells were obtained from the Cell Resource Center for Biomedical Research. Cells were maintained at 37 °C and 5% CO_2_ in Dulbecco's modified Eagle's medium supplemented with 10% fetal bovine serum, 100 units/ml penicillin, and 100 μg/ml streptomycin, as described previously (20).

### Preparation of plasmids

The complementary DNAs (cDNAs) of hSLC19A2 and hSLC19A3 (GenBank accession numbers: NM_006996.3 and NM_025243.4, respectively) were prepared as described previously (1). The cDNA of pig SLC19A2 (GenBank accession number: NM_001142667.1) and cDNAs of SLC19A3s of rabbit, guinea pig, and hamster (GenBank accession numbers: XM_008259270.2, XM_013157741.2, and XM_005082525.4, respectively) were obtained as synthetic products from Integrated DNA Technologies.

The cDNA of monkey SLC19A2 was cloned using an RT–PCR method, as described previously (20). Briefly, an RT reaction was carried out to obtain a cDNA mixture from monkey small intestine total RNA (UNITEC) using 1 μg of total RNA, an oligo(dT) primer, and ReverTra Ace (Toyobo) as a reverse transcriptase. The cDNA of SLC19A2 was amplified by PCR using KOD One Polymerase (Toyobo). Then, a second PCR was performed using the amplified product as a template to incorporate restriction sites. The GenBank accession number for the cDNA and the primers for PCR are shown in Table S1.

The cDNAs of the other SLC19A2 and SLC19A3 orthologs were cloned similarly, using the total RNA samples and PCR primers shown in Table S1. Their GenBank accession numbers are shown in Table S1. The total RNA samples of dog, pig, and rat were prepared from Madin–Darby canine kidney II cells, Lilly Laboratories cell-porcine kidney 1 cells, and the rat small intestine, respectively, by a guanidine isothiocyanate extraction method (21). The rat small intestine total RNA was prepared using male Wistar rats with the approval of the Animal Experimental Ethics Committee of Nagoya City University Graduate School of Pharmaceutical Sciences. The total RNA samples of mouse and frog were obtained from BioChain.

The cDNAs for the mutants, in which single or multiple amino acid residues were replaced in hSLC19A2, hSLC19A3, and mSlc19a3, were generated by a site-directed mutagenesis method (PrimeSTAR Mutagenesis Basal Kit; Takara Bio), using KOD One Polymerase. The primers for PCR are shown in Table S2.

All final cDNA products for the SLC19A2 and SLC19A3 orthologs were incorporated into pCI-neo vector (Promega) to prepare plasmids for transfection, and their sequences were determined with an automated sequencer (ABI PRISM 3130; Applied Biosystems), as described previously (22). For hSLC19A2, hSLC19A3, mSlc19a3, and mutants, plasmids using pEGFP-C1 vector (Promega) were prepared similarly. The enhanced GFP (EGFP)–tagged transporters were used for routinely inspecting expression levels in transiently transfected HEK293 cells using fluorescence microscopy.

### Preparation of transiently transfected HEK293 cells

HEK293 cells (2.0 × 10^5^ cells/ml, 1 ml/well) were grown on 24-well plates coated with poly-l-lysine for 12 h, transfected with 1 μg/well of the plasmid carrying the cDNA of the designated transporter or mutant using 2 μg/well of polyethylenimine “MAX” (Polyscience), and cultured for 48 h for transient expression, as described previously (22). Mock cells were prepared similarly using empty pCI-neo or pEGFP-C1 vector.

### Uptake assays

Uptake assays were conducted as described previously (1, 20), using transiently transfected HEK293 cells cultured on 24-well plates. Briefly, uptake solutions were prepared using Hanks' solution modified by supplementation with 10 mM Mes (pH 5.5) for [^3^H]pyridoxine as a substrate or 10 mM Hepes (pH 7.4) for [^3^H]thiamine as a substrate. Cells in each well were preincubated for 5 min in 1 ml of substrate-free uptake solution. Uptake assays were started by replacing the substrate-free uptake solution with 0.25 ml of uptake solution containing a substrate. All procedures were conducted at 37 °C. After termination of substrate uptake into the cells, the cells were solubilized, and the associated radioactivity was determined by liquid scintillation counting for the evaluation of the uptake. The uptake was normalized to cellular protein content, which was determined by the bicinchoninic acid method (BCA Protein Assay Reagent Kit; Thermo Fisher Scientific) using bovine serum albumin as the standard. Uptake assays were also conducted in mock cells transfected with empty pCI-neo or pEGFP-C1 vector to estimate nonspecific uptake. The specific uptake was estimated by subtracting the nonspecific uptake from the uptake in transfected cells.

### Statistical analysis

Data are presented as means ± SD, with the number of experiments conducted using different preparations of cells. Each experiment was conducted in duplicate as biological repeats. Statistical analysis was performed by using ANOVA followed by Dunnet's test, with *p* < 0.05 considered significant.

## Data availability

All data are contained within the article.

## Supporting information

This article contains supporting information.

## Conflict of interest

The authors declare that they have no conflicts of interest with the contents of this article.
