# Supplementary material for: Identification of the amino acid residues involved in the species-dependent differences in the pyridoxine transport function of SLC19A3
Source: J Biol Chem. 2022 Jun 17;298(8):102161. doi: 10.1016/j.jbc.2022.102161 (PMC9293782; doi:10.1016/j.jbc.2022.102161)
Supplement: Supporting information [file mmc1.docx]

SUPPORTING INFORMATION

**Identification of the amino acid residues involved in the animal species differences in the pyridoxine transport function of SLC19A3**

**Authors:**

Kohei Miyake, Tomoya Yasujima, Syunsuke Takahashi, Takahiro Yamashiro, Hiroaki Yuasa

**Affiliations:**

*Department of Biopharmaceutics, Graduate School of Pharmaceutical Sciences, Nagoya City University, 3-1 Tanabe-dori, Mizuho-ku, Nagoya 467-8603, Japan*

**Table S1**

**Total RNA samples and PCR primers for preparation of the cDNAs of SLC19A2 and SLC19A3 orthologs**

Underlined with solid, broken, double, and bold lines are restriction sites for EcoRI, SalI, XhoI, and XbaI, respectively.

| Transporter | Species | GenBank accession No. | Source of total RNA | PCR | Direction | Sequence (5’ to 3’) |
| --- | --- | --- | --- | --- | --- | --- |
| SLC19A2 | Monkey | XM_005539926.2 | Small intestine from cynomolgus monkey | 1st | Forward  Reverse | AGTGCCTGACCCTCATTCCAGTTG  AATCAAATACATCCAGGCAGTTGCT |
|  |  |  |  | 2nd | Forward  Reverse | CCTGAATTCCGCCATGGATGTGCCCGGCCCG  AGCGTCGACTTATGAAGTGGTTACTTG |
|  | Dog | XM_038671431.1 | MDCKII cells | 1st | Forward  Reverse | TCCCTCCCGCGAATGGATGTG  AAATACATCCGGGCACAGTTGCT |
|  |  |  |  | 2nd | Forward  Reverse | TCCCTCGAGGGAATGGATGTGCCCGGCCCG  AGCTCTAGAATCATGAAGTGGTTACATG |
|  | Rat | NM_001030024.2 | Small intestine from  Wistar rat | 1st | Forward  Reverse | GACTCCAAGCACCTTGACCCT  AGCCACAGAGAGTGTAAGTACATCC |
|  |  |  |  | 2nd | Forward  Reverse | GTCCTCGAGAGGATGGACGTGCCCGCCCGG  GTTTCTAGATTAGGAAGTGGTTGCTTG |
|  | Mouse | NM_054087.3 | Small intestine from  A/J mouse | 1st | Forward  Reverse | GACTCCAAGCACCTTGACCCTG  CGCACAAGTAACACTGAAACCAGA |
|  |  |  |  | 2nd | Forward  Reverse | GGACTCGAGAGGATGGACGTGCCAGCCAGG  GTTTCTAGATTAGGACGTGGAGGCTTG |
|  | Frog | OCT91659.1 | Small intestine from  African clawed frog | 1st | Forward  Reverse | GGCAACAACTAAATGCACTGA  CAGATGTTTAATATCGGCCCTG |
|  |  |  |  | 2nd | Forward  Reverse | CATCTCGAGGCCATGGAGATGAATGGGGC  GTATCTAGATTACTTCAAGGATCCGATG |
| SLC19A3 | Monkey | XM_015432908.1 | Small intestine from cynomolgus monkey | 1st | Forward  Reverse | ATCAGCATGAAGTAGTTTTGCCT  CTTTAAAAGCCACTGCTGCAAT |
|  |  |  |  | 2nd | Forward  Reverse | GAGGAATTCTGCCATGGATTGTTACAGAA  TTGTCTAGAGGTTAGAGTTTTGTTGAC |
|  | Dog | XM_014107609.3 | MDCKII cells | 1st | Forward  Reverse | AGCTTTATTGAGCAACTACTATGTGCCT  CGGTCTAAAACTGAGATTTTGTTGTGGT |
|  |  |  |  | 2nd | Forward  Reverse | GGCGAATTCAGCCATGGATTGTTTCAA  GCCTCTAGATTAGAGCTTTGTTCCTGT |
|  | Pig | XM_021075295.1 | LLC-PK1 cells | 1st | Forward  Reverse | TCAAGGAAGAAATAAGTTCGGGACCCA  GTGATTCTATGACCAGCACAGTCCT |
|  |  |  |  | 2nd | Forward  Reverse | GAGCTCGAGGCCATGAGTTGTTTCCAA  GTTTCTAGAGGTTCATCAGACATTCTT |
|  | Rat | NM_001108228.1 | Small intestine from  Wistar rat | 1st | Forward  Reverse | TCTTTACTTGAGATCGCGGACCAC  AGTCGATGTAACTGGGTTAGAACT |
|  |  |  |  | 2nd | Forward  Reverse | GACGAATTCTGCCATGAATTGCTGCAGAA  TCGTCTAGACTGGGTTAGAACTTCGTTG |
|  | Mouse | NM_030556.2 | Small intestine from  A/J mouse | 1st | Forward  Reverse | GCTTAATCAACTAGCATCGTGGGA  TCTTAAAATCCTGCCCATTTGTGAC |
|  |  |  |  | 2nd | Forward  Reverse | GACGAATTCCGCCATGGACTCCAGCTGCAG  GATGTCGACGGGTTAGAACTTTGTTGACA |
|  | Frog | XM_018263940.2 | Small intestine from  African clawed frog | 1st | Forward  Reverse | AGGTTTTAACATCATCCGTTTGGC  ACTTCCCCTCTGTACTGTGC |
|  |  |  |  | 2nd | Forward  Reverse | TCGGAATTCAAACATGGATATTCCTGGCT  CCTTCTAGATCTAAAATTGTGTCTGCTCC |

**Table S2**

**Primers for generation of the cDNAs of SLC19A2 and SLC19A3 mutants**

| Transporter | Mutant | Direction | Sequence (5’ to 3’) |
| --- | --- | --- | --- |
| hSLC19A3 | mTMD1 | Forward  Reverse | CACTGTGATCCTCTGCTTATTTGGTTTTTTCTCCATGTTTAGACCCTCAGAACCATTCCT  CAGAGGATCACAGTGGGGTAAACCCAGGAATTGCTTGGTGGAGTTCTGTAACAATCCAT |
|  | mTMD2 | Forward  Reverse | ACATACTCCTACCTGGCGACGCTGCCGCCTGTGTTTGTCCTCACCG  CAGGTAGGAGTATGTCCAAACGGGCAAGATCTCATTTGTTATCTC |
|  | mTMD3 | Forward  Reverse | TCGCTTTCGCCACTTCCTACCTGTTCCTGTTGTTTGGCCAAGGAGT  AAGTGGCGAAAGCGACAACATGCAACATGATGACTGGCTTGTAGCG |
|  | mTMD4 | Forward  Reverse | CAGCGCCACCGAGATTGCCTACTTCGCCTACATATACAGCATGGTCAGCCCCGAGCACTA  ATCTCGGTGGCGCTGACCACCCCAAAGAAGAACTCTGCAGTCTGCATGGTCTTCACTCCT |
|  | mTMD5 | Forward  Reverse | GCATCACGCTGGTCGCCTACACAGCAGGGTCGG  CGACCAGCGTGATGCTCCTGCAGTAGCCGCTCA |
|  | mTMD6 | Forward  Reverse | TACATATCCTTGGCCTGTGTCTCCGTGGCTTTCTTTTTCTCACTTTTCCTACCA  GGCCAAGGATATGTAGAAGAGGGAAGAGTACGGCATGTTCGCCAGGGATACCA |
|  | mTMD7 | Forward  Reverse | AGATTTTGAACTATGTTCAAGTCCTGTGGGATTACAAGGCG  CATAGTTCAAAATCTGGTTATAACCTGCTGTGGCGAAAGCC |
|  | mTMD8 | Forward  Reverse | TTGGCTTCCTTTTCAGTGGGTTATGTGAAAGTC  TGAAAAGGAAGCCAAAGCCCCTCCAAAGGTTGCAA |
|  | mTMD9 | Forward  Reverse | AGCTGTCATTGCCGGTTCTTTATTTCTCATGAATTACACAGCCAATATCTGG  CCGGCAATGACAGCTGAGAAGACCGCCAGACCCAGCTCTCCCAGAAGGTCC |
|  | mTMD10 | Forward  Reverse | CTATTTGCTAGTCAAGTCCAGCTATTCGTTTCTTATAACCATAGCAGTA  TTGACTAGCAAATAGCCAGCATAGCACACCCAGATATTGGCTGTGTAA |
|  | mTMD11 | Forward  Reverse | GCCTTGGTGATTCAGACCATCATGACTATGATTGTAGTAGATCAGAGA  CTGAATCACCAAGGCAATAAAGGTGTCGATTCCAAATACCAAGGCAT |
|  | mTMD12 | Forward  Reverse | TTATGGGAGCTATTTTGCAGTAATTGCTGGAGTTTTCCTAATGAGAAGCATG  AAAATAGCTCCCATAAACTAAAAACTGAGTGGTGACTGGCAAGTTGAGCCCT |
|  | I84M | Forward  Reverse | AGTCATCATGTTGCAAGGTATCAGTTTC  CTTGCAACATGATGACTGGCTTGTAGCG |
|  | Q86H | Forward  Reverse | CATCTTGCACGGTATCAGTTTCATCATT  TGATACCGTGCAAGATGATGACTGGCTT |
|  | G87V | Forward  Reverse | CTTGCAAGTTATCAGTTTCATCATTACC  AACTGATAACTTGCAAGATGATGACTGG |
|  | I88V | Forward  Reverse | GCAAGGTGTGAGTTTCATCATTACCTGG  TGAAACTCACACCTTGCAAGATGATGAC |
|  | S89A | Forward  Reverse | AGGTATCGCCTTCATCATTACCTGGCTG  TGATGAAGGCGATACCTTGCAAGATGAT |
|  | I91A | Forward  Reverse | CAGTTTCGCCATTACCTGGCTGCTGCTG  AGGTAATGGCGAAACTGATACCTTGCAA |
|  | I92T | Forward  Reverse | TTTCATCACTACCTGGCTGCTGCTGTTG  GCCAGGTAGTGATGAAACTGATACCTTG |
|  | T93S | Forward  Reverse | CATCATTAGCTGGCTGCTGCTGTTGTTT  GCAGCCAGCTAATGATGAAACTGATACC |
|  | W94Y | Forward  Reverse | CATTACCTACCTGCTGCTGTTGTTTGGC  GCAGCAGGTAGGTAATGATGAAACTGAT |
|  | L96F | Forward  Reverse | CTGGCTGTTTCTGTTGTTTGGCCAAGGA  ACAACAGAAACAGCCAGGTAATGATGAA |
|  | V108T | Forward  Reverse | CATGCAGACTGTAGAGTTCTTCTATGG  CTCTACAGTCTGCATGGTCTTCACTCC |
|  | V109A | Forward  Reverse | CAGGTTGCAGAGTTCTTCTATGGGAT  GAACTCTGCAACCTGCATGGTCTTCA |

To be continued.

**Table S2**

Continued.

| Transporter | Mutant | Direction | Sequence (5’ to 3’) |
| --- | --- | --- | --- |
| hSLC19A3 | Y113F | Forward  Reverse | GTTCTTTTTTGGGATGGTCACCGCCGCC  CCATCCCAAAAAAGAACTCTACAACTTG |
|  | M115V | Forward  Reverse | TTATGGGGTGGTCACCGCCGCCGAGGTG  CGGTGACCACCCCATAAAAGAACTCTAC |
|  | T117S | Forward  Reverse | GATGGTCTCCGCCGCCGAGGTGGCCTAC  CGGCGGCGGAGACCATCCCATAAAAGAA |
|  | A119T | Forward  Reverse | CACCGCCACCGAGGTGGCCTACTACGCC  CCACCTCGGTGGCGGTGACCATCCCATA |
|  | V121I | Forward  Reverse | CGCCGAGATAGCCTACTACGCCTACATA  AGTAGGCTATCTCGGCGGCGGTGACCAT |
|  | Y124F | Forward  Reverse | GGCCTACTTCGCCTACATATACAGCGTG  TGTAGGCGAAGTAGGCCACCTCGGCGGC |
|  | V130M | Forward  Reverse | ATACAGCATGGTCAGCCCCGAGCACT  CTGACCATGCTGTATATGTAGGCGTA |
|  | M167L | Forward  Reverse | GGCGAACCTGTCGTACTTTTACCTCAAC  AGTACGACAGGTTCGCCAGGGATACCAA |
|  | S168P | Forward  Reverse | GAACATGCCATACTTTTACCTCAACGTC  AAAAGTATGGCATGTTCGCCAGGGATAC |
|  | F170S | Forward  Reverse | GTCGTACTCTTACCTCAACGTCATATCC  TGAGGTAAGAGTACGACATGTTCGCCAG |
|  | Y171S | Forward  Reverse | GTACTTTAGCCTCAACGTCATATCCTTG  CGTTGAGGCTAAAGTACGACATGTTCGC |
|  | N173F | Forward  Reverse | TTACCTCTTTGTCATATCCTTGGCCTCT  ATATGACAAAGAGGTAAAAGTACGACAT |
|  | V174Y | Forward  Reverse | CCTCAACTATATATCCTTGGCCTCTGTC  AGGATATATAGTTGAGGTAAAAGTACGA |
|  | S179C | Forward  Reverse | CTTGGCCTGTGTCTCCGTGGCTTTCCTT  CGGAGACACAGGCCAAGGATATGACGTT |
|  | L185F | Forward  Reverse | GGCTTTCTTTTTCTCACTTTTCCTACCA  GTGAGAAAAAGAAAGCCACGGAGACAGA |
| mSlc19a3 | hA3-5AA | Forward  Reverse | GCTACACGGTGTGGCCTTCGCCACTACCTGGCTGTTTCTCTTGTTTGGCCA  GAAACAGCCAGGTAGTGGCGAAGGCCACACCGTGTAGCATGATGACTGGTT |
|  | hTMD3 | Forward  Reverse | GTATCAGTTTCATCATTACCTGGCTGCTGCTCTTGTTTGGCCAAGGTGT  AATGATGAAACTGATACCTTGTAGGATGATGACTGGTTTGTAGCGCA |
|  | hTMD4 | Forward  Reverse | CCGAGGTGGCCTACTACGCCTACATATACAGCGTGGTCAGCCCAGAACACTATCA  GGCGGTGACCATCCCATAAAAGAACTCTACAACTTGCATGAGCATCACACCTT |
|  | hTMD6 | Forward  Reverse | TTTTACCTTAACGTCATATCCTTGGCCTCTGTCTCTGTGGCTTTCCTTTTCTCACTTTTTCTACCAAT  TCCTGGTATCCCTGACGAACATGTCGTACTTTTACCTTAACGTCATA |
|  | hTMD3+4: primers for hTMD3 and hTMD4 together | | |
|  | hTMD3+6: primers for hTMD3 and hTMD6 together | | |
|  | hTMD4+6: primers for hTMD4 and hTMD6 together | | |
|  | hTMD3+4+6: primers for hTMD3, hTMD4, and hTMD6 altogether | | |

To be continued.

**Table S2**

Continued.

| Transporter | Mutant | Direction | Sequence (5’ to 3’) |
| --- | --- | --- | --- |
| mSlc19a3-hTMD3+6 | I85M | Forward  Reverse | AGTCATCATGTTGCAAGGTATCAGTTTC  CTTGCAACATGATGACTGGCTTGTAGCG |
|  | Q87H | Forward  Reverse | CATCTTGCACGGTATCAGTTTCATCATT  TGATACCGTGCAAGATGATGACTGGCTT |
|  | G88V | Forward  Reverse | CTTGCAAGTTATCAGTTTCATCATTACC  AACTGATAACTTGCAAGATGATGACTGG |
|  | I89V | Forward  Reverse | GCAAGGTGTGAGTTTCATCATTACCTGG  TGAAACTCACACCTTGCAAGATGATGAC |
|  | S90A | Forward  Reverse | AGGTATCGCCTTCATCATTACCTGGCTG  TGATGAAGGCGATACCTTGTAGGATGAT |
|  | I92A | Forward  Reverse | CAGTTTCGCCATTACCTGGCTGCTGCTG  AGGTAATGGCGAAACTGATACCTTGCAA |
|  | I93T | Forward  Reverse | TTTCATCACTACCTGGCTGCTGCTGTTG  GCCAGGTAGTGATGAAACTGATACCTTG |
|  | T94S | Forward  Reverse | CATCATTAGCTGGCTGCTGCTGTTGTTT  GCAGCCAGCTAATGATGAAACTGATACC |
|  | W95Y | Forward  Reverse | CATTACCTACCTGCTGCTGTTGTTTGGC  GCAGCAGGTAGGTAATGATGAAACTGAT |
|  | L97F | Forward  Reverse | CTGGCTGTTTCTGTTGTTTGGCCAAGGA  ACAACAGAAACAGCCAGGTAATGATGAA |
|  | M168L | Forward  Reverse | GACGAACCTGTCGTACTTTTACCTTAAC  AGTACGACAGGTTCGTCAGGGATACCAG |
|  | S169P | Forward  Reverse | GAACATGCCATACTTTTACCTCAACGTC  AAAAGTATGGCATGTTCGTCAGGGATAC |
|  | F171S | Forward  Reverse | GTCGTACTCTTACCTTAACGTCATATCC  TAAGGTAAGAGTACGACATGTTCGTCAG |
|  | Y172S | Forward  Reverse | GTACTTTAGCCTTAACGTCATATCCTTG  CGTTAAGGCTAAAGTACGACATGTTCGT |
|  | N174F | Forward  Reverse | TTACCTCTTTGTCATATCCTTGGCCTCT  ATATGACAAAGAGGTAAAAGTACGACAT |
|  | V175Y | Forward  Reverse | CCTCAACTATATATCCTTGGCCTCTGTC  AGGATATATAGTTGAGGTAAAAGTACGA |
|  | S180C | Forward  Reverse | CTTGGCCTGTGTCTCCGTGGCTTTCCTT  CGGAGACACAGGCCAAGGATATGACGTT |
|  | L186F | Forward  Reverse | GGCTTTCTTTTTCTCACTTTTCCTACCA  GTGAGAAAAAGAAAGCCACGGAGACAGA |
| mSlc19a3 | hA3-7AA | Forward  Reverse | GTGGCCTTCGCCACTACCTGGCTGTTTCTCTTGTTTGGC  AGTGGCGAAGGCCACACCTTGTAGCATGATGACTGGTTT |
|  |  | Forward  Reverse | CGTACTCTAGCCTTAACTATATATCCTTGGCCTGT  TTAAGGCTAGAGTACGACAGGTTCGTCAGGGATAC |
| hSLC19A2 | ma3-7AA | Forward  Reverse | TTCTACTGCACGTTCTCAGCCTTGCCGTTAGCTACTTTATGCTGCTCTATGCC  GGCATAGAGCAGCATAAAGTAGCTAACGGCAAGGCTGAGAACGTGCAGTAGAA |
|  |  | Forward  Reverse | CAGGCTGGTCGCTGTTCAGCCTGAATGTCATCTCTCTTACCTGT  ACAGGTAAGAGAGATGACATTCAGGCTGAACAGCGACCAGCCTG |
